# The Potential Mechanisms of Ochratoxin A in Prostate Cancer Development: An Integrated Study Combining Network Toxicology, Machine Learning, and Molecular Docking

**DOI:** 10.3390/toxins17080388

**Published:** 2025-08-04

**Authors:** Hong Cai, Dandan Shen, Xiangjun Hu, Hongwei Yin, Zhangren Yan

**Affiliations:** College of Clinical Medicine, Jiangxi University of Chinese Medicine, Wanli Campus, Nanchang 330004, China; cai_hong0130@163.com (H.C.); jellycatpure@163.com (D.S.); hxj199625@163.com (X.H.); yinhongweijxszyy@163.com (H.Y.)

**Keywords:** ochratoxin A, prostate cancer, network toxicology, molecular docking, machine learning, environmental carcinogenesis

## Abstract

Ochratoxin A (OTA), a prevalent food contaminant, has been proposed as a potential contributor to the development of prostate cancer, although its precise mechanisms remain unclear. This study employed a comprehensive approach that integrated network toxicology, machine learning, and molecular docking to clarify the role of OTA in prostate cancer. The findings indicated that OTA interacts with 364 targets related to prostate cancer, and machine learning was employed to identify five key molecular targets as priorities (ESR1, TP53, TNF, INS, and EGFR). In conjunction with the results of a functional enrichment analysis, OTA was found to possibly facilitate cancer progression by disrupting endocrine function, activating oncogenic signaling pathways, reprogramming metabolism, and modulating the tumor microenvironment.

## 1. Introduction

In recent years, due to the globalization of the food supply chain, the issue of mycotoxin contamination has become increasingly widespread, posing a significant challenge to public health [1]. Mycotoxins are secondary metabolites produced by filamentous fungi, such as Aspergillus and Penicillium species, which continuously contaminate agricultural products and the environment, posing a severe threat to human health [2]. Among these toxins, ochratoxin A (OTA) is particularly concerning. OTA is a heat-stable mycotoxin commonly found in grains, coffee, and nuts. According to monitoring data from the European Food Safety Authority, this toxin is detected in EU grain samples at a rate exceeding 30% [3]. Its phenylalanine derivative structure confers significant tolerance to processing, allowing it to remain highly active even after high-temperature treatment [4]. This characteristic means that OTA may persist in thermally processed foods, such as roasted coffee and baked grain products [5,6].

The International Agency for Research on Cancer (IARC) classifies ochratoxin A (OTA) as a Group 2B possible human carcinogen, with this classification primarily based on evidence from animal studies [7]. OTA has been shown to cause multi-organ toxicity, affecting the kidneys, liver, nervous system, and immune system and potentially causing birth defects and genetic damage in various animal models [8]. 

Prostate cancer is the second most commonly diagnosed malignancy among men worldwide. Its development is influenced by a complex interplay of genetic predisposition, hormonal signals, and exposure to environmental carcinogens [9,10]. Research indicates that dietary factors, including exposure to foodborne toxins, are linked to the incidence of prostate cancer [11,12]. Recent evidence suggests that ochratoxin A (OTA) might also affect prostate biology through additional mechanisms, such as mitochondrial dysfunction [13], epigenetic changes [14], and disruptions to the gut–urogenital microbiome [15]. However, evidence supporting its carcinogenic potential is still insufficient, and the specific role of OTA in the development of prostate cancer has been relatively under-researched.

To further investigate the potential relationship between ochratoxin A (OTA) and prostate cancer (PCa), this study integrates network toxicology, machine learning, and molecular docking to systematically uncover the potential connection between OTA and PCa. Network toxicology is an interdisciplinary research approach that combines systems biology with computational toxicology, creating multi-level network models consisting of “compounds–targets–pathways–diseases” to clarify the potential toxic effects of chemicals on biological systems [16]. Machine learning involves the automatic identification of patterns within data through algorithms, is used for predictive modeling and decision making, and is effective in extracting key biomarkers from complex datasets [17]. Molecular docking is a computational technique that simulates the binding of molecules to estimate free energies and predict the geometries of ligand–protein interactions, thereby supporting hypotheses generated through network toxicology [18]. Initially, we constructed a protein–protein interaction (PPI) network and conducted a functional enrichment analysis using network toxicology methodologies. Then, we applied machine learning algorithms to pinpoint core targets and utilized molecular docking to forecast the binding conformations and affinities between OTA and these key targets, thereby elucidating its potential carcinogenic mechanisms.

Our preliminary findings may offer clues for exploring the potential link between ochratoxin A and prostate cancer, laying a foundation for future experimental validation and strategies aimed at preventing dietary exposure to mycotoxins.

## 2. Results

### 2.1. Toxicity Model Computation of Ochratoxin A

**Figure 1:**
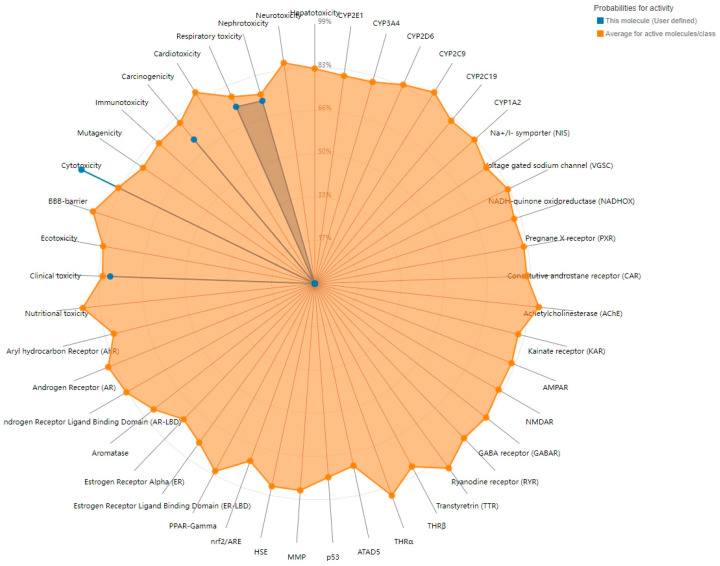
Computational model of OTA toxicity.

The results from the ProTox-3.0 toxicity prediction model indicate that OTA exhibits significant carcinogenicity (probability 0.71) and nephrotoxicity (probability 0.72) (Figure 1, Table 1) and that OTA has extremely high cytotoxicity (probability 0.99) and clinical toxicity (probability 0.78).

### 2.2. Targets of Ochratoxin A in Prostate Cancer

**Figure 2:**
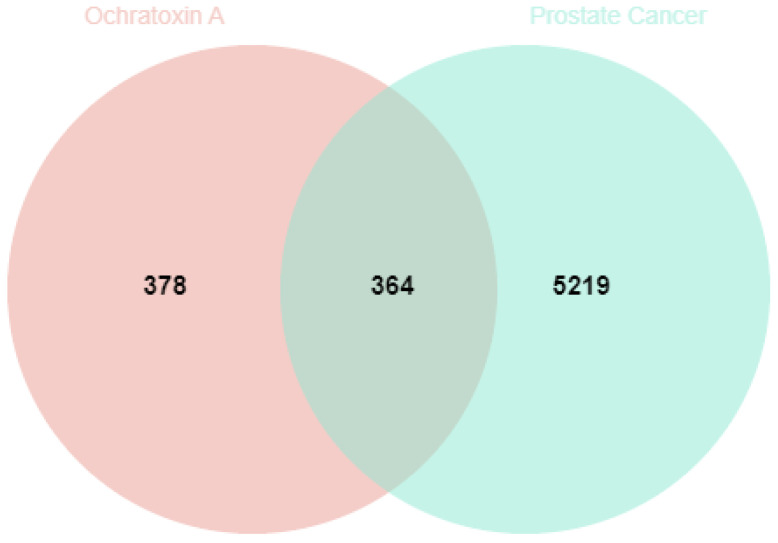
Venn diagram of OTA and PCa targets.

We first obtained 742 targets of OTA from the ChEMBL and SwissTargetPrediction target prediction databases. Subsequently, we identified 5583 targets highly associated with prostate cancer from the GeneCards database. After integrating these target sets and removing duplicates, a total of 364 overlapping targets were identified. To evaluate whether this overlap exceeded random expectation, we performed a hypergeometric test (N = 19,871 (total annotated human protein-coding genes in Ensembl release 114/GRCh38.p14); K = 742 OTA targets; and M = 5583 prostate cancer genes). The test yielded an extremely significant *p*-value < 1.0 × 10^−50^. These 364 overlapping genes are, therefore, considered high-confidence potential targets through which OTA may promote prostate carcinogenesis (Figure 2).

### 2.3. Protein–Protein Interaction Network of Common Targets

**Figure 3:**
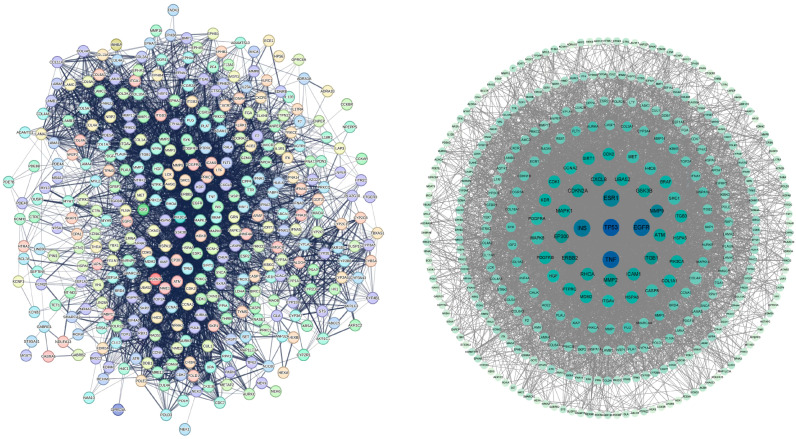
PPI network of shared targets between OTA and PCa.

We constructed a protein–protein interaction (PPI) network for the 364 common targets using the STRING database. The topological properties of the network nodes were then analyzed using Cytoscape software (version 3.9.1) (Figure 3).

### 2.4. GO Functional and KEGG Pathway Enrichment Analyses

**Figure 4:**
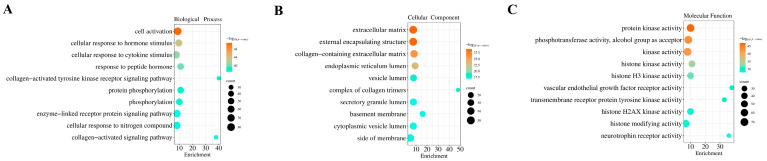
GO functional analysis of OTA-induced PCa (top 10 terms). (**A**) Biological processes; (**B**) cellular components; and (**C**) molecular functions. The size of each bubble corresponds to the number of genes associated with each functional term, and the darker the bubble color, the more significant the functional term.

We performed GO functional and KEGG pathway enrichment analyses on the 364 targets using the Metascape database. A total of 3111 GO terms were obtained from the GO functional enrichment analysis, including 2534 biological processes (BPs), 218 cellular components (CCs), and 359 molecular functions (MFs). The BPs were mainly involved in cell activation and signal response, collagen-related signaling pathways, and the regulation of protein phosphorylation. The CCs were primarily associated with extracellular matrix (ECM)-related structures, the endoplasmic reticulum lumen, and the vesicle lumen. The MFs were mainly related to protein kinase, histone modification, and phosphotransferase activities. The top 10 enriched BPs, CCs, and MFs were selected for visualization (Figure 4).

**Figure 5:**
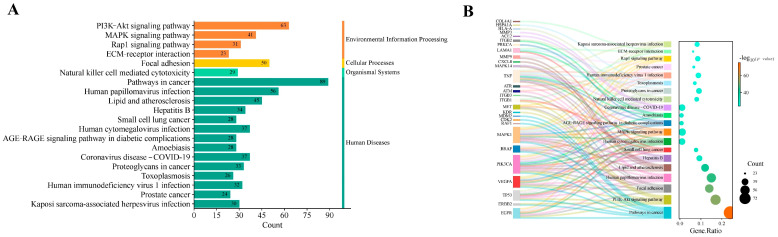
KEGG pathway enrichment analysis of OTA-induced PCa (top 20 terms). (**A**) Histogram showing the number of enriched pathways and (**B**) Sankey diagram illustrating the associations between targets and pathways. The size of each bubble corresponds to the number of genes associated with each functional term, and the darker the bubble color, the more significant the functional term.

The KEGG pathway enrichment analysis results (top 20 terms) indicated that after OTA treatment, the main signaling pathways active in cells are those primarily involved in environmental information processing, including the PI3K/Akt, MAPK, and Rap1 signaling pathways and ECM–receptor interaction. Additionally, cell adhesion is mainly involved in cellular processes, while pathways such as natural-killer-cell-mediated cytotoxicity, cancer-related pathways, human papillomavirus infection, and the AGE-RAGE signaling pathway are implicated within the whole organismal system, with the latter involved primarily in diabetic complications. In the context of human diseases, OTA may affect the pathway related to oncoproteoglycan [19], but its potential interaction with coronavirus disease (COVID-19) and amoebiasis-related pathways lacks direct evidence, supported only by theoretical speculation. OTA may promote the occurrence and development of prostate cancer by activating key mechanisms such as the PI3K/Akt signaling pathway, thereby affecting the extracellular matrix, metabolism, and immune function. Additionally, OTA may interact with various viral infections and metabolic disorders (Figure 5).

### 2.5. Identification of Potential Core Targets

**Figure 6:**
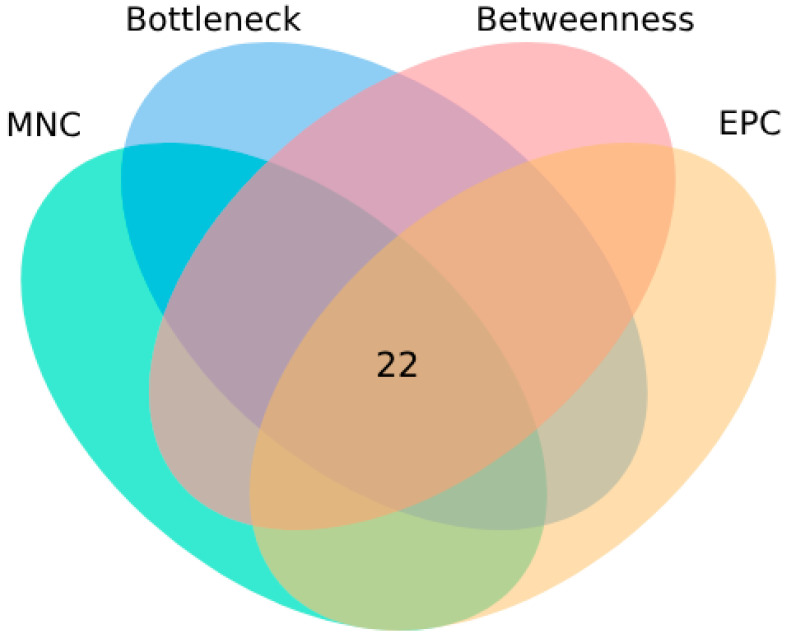
Venn diagram of intersection targets identified using four algorithms.

**Figure 7:**
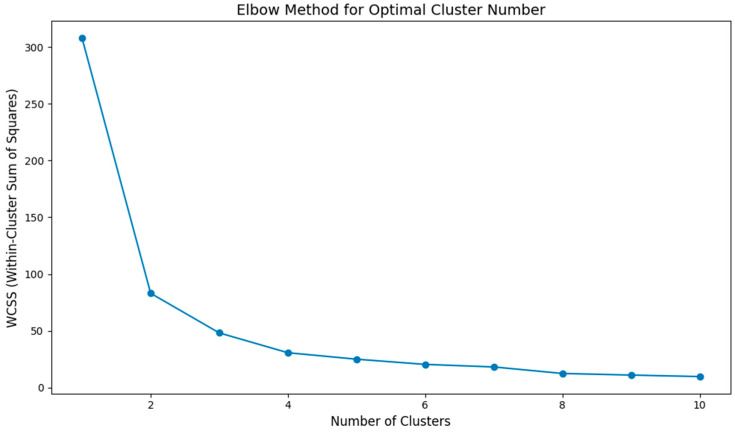
Elbow plot for K-means clustering analysis.

We utilized four algorithms (MNC, EPC, Bottleneck, and Betweenness) in Cytoscape software to identify 22 intersecting targets (Figure 6). Subsequently, the elbow method was employed to determine the optimal number of clusters for the K-means clustering analysis. By calculating the within-cluster sum of squares (WCSS) for different numbers of clusters, we observed that the rate of decrease in WCSS significantly slowed when the number of clusters reached three (Figure 7). This indicated that three clusters could effectively balance the compactness and separation of the clustering. Based on this result, we fixed the number of clusters for K-means at three and further performed clustering on the standardized feature matrix.

**Figure 8:**
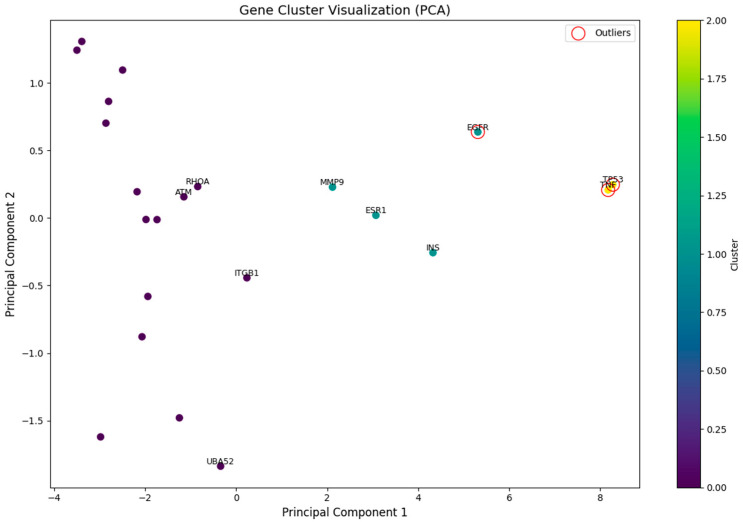
PCA-based visualization of K-means clustering (k = 3) for 22 genes. The colors represent three clusters determined via K-means; the red circles denote outliers identified using the Isolation Forest algorithm.

Next, we performed K-means clustering on the normalized feature data and applied a principal component analysis (PCA) to reduce the high-dimensional data to a two-dimensional space for visualization (Figure 8). In the PCA-reduced gene clustering plot, different colors represent different clusters, while points marked with red circles indicate genes identified as outliers by the Isolation Forest algorithm. These outlier genes are distinctly separated from others in the principal component space, suggesting unique characteristics in the original feature space and potentially significant biological implications.

Additionally, we calculated the Z-score-normalized values and percentile ranks of various network topology metrics to construct a composite score (Composite_score) for evaluating gene importance. A lower composite score indicates a higher importance of a gene within the network. This approach enabled us to identify genes with potential key roles in the network. The results showed that the top 10 genes (Table 2) exhibited prominent performances across multiple topology metrics, suggesting their potential functional or regulatory significance. Notably, TP53 and TNF had the lowest composite scores (4.14 and 4.71, respectively), indicating their highest importance in the network. TP53, TNF, and EGFR were marked with red circles, suggesting their potential special significance in the pathogenesis of prostate cancer.

### 2.6. Molecular Docking Results of Core Targets of OTA in PCa

**Figure 9:**
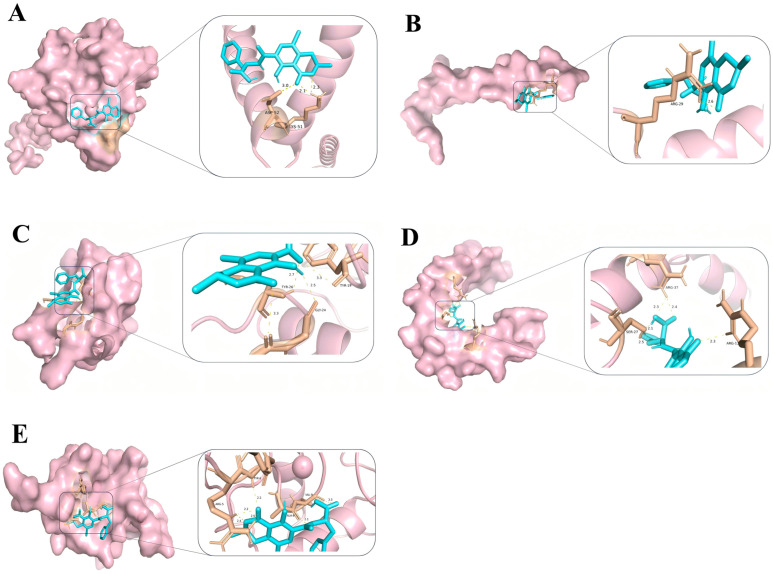
Molecular docking diagrams of OTA with the top five targets. (**A**) Docking results of OTA with TP53; (**B**) docking results of OTA with TNF; (**C**) docking results of OTA with INS; (**D**) docking results of OTA with EGFR; and (**E**) docking results of OTA with ESR1.

**Figure 10:**
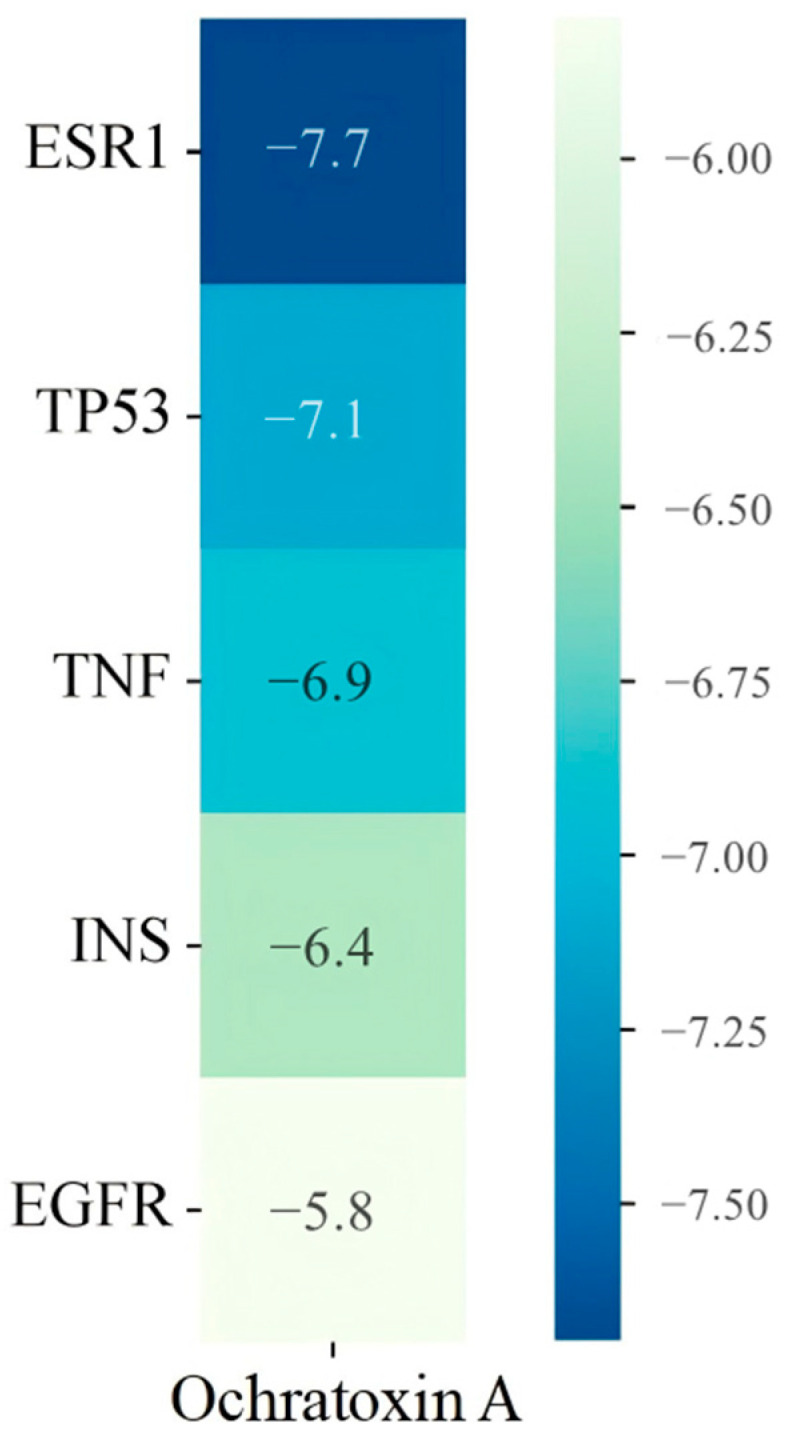
Binding energy heatmap of OTA with the top five targets (kcal/mol).

We selected the top five targets based on their composite ranking for molecular docking (Figure 9). Molecular docking was performed to validate the interactions between OTA and these core targets, and the binding affinities were analyzed based on their binding energies (Figure 10). A lower binding energy indicates a more stable interaction between the ligand and the receptor, suggesting a higher likelihood of interaction [20]. The molecular docking results revealed that ESR1 (an estrogen receptor) and TP53 (a tumor suppressor gene) had the strongest binding energies (≤−7.0 kcal/mol), indicating that OTA may significantly affect the functions of these targets. TNF (inflammatory cytokine) and INS (insulin) also exhibited favorable binding energies (≤−6.5 kcal/mol), suggesting their potential involvement in inflammation or metabolic regulation. Although the binding energy of EGFR was relatively weaker, as an important cell surface receptor, its interaction with OTA may still influence cell signaling and proliferation. According to the literature, a binding energy that is less than or equal to −7.0 kcal/mol corresponds to affinity ranging from nM to μM, suggesting that OTA may directly interfere with the functions of these target proteins. Furthermore, a binding energy ranging from −5.0 to −7.0 kcal/mol may require the synergistic action of multiple targets [21,22,23,24]. Natural products (such as ochratoxin A) often modulate complex disease networks through such moderate binding interactions, which is consistent with our findings [25].

## 3. Discussion

This study utilized an integrated approach that combined network toxicology, machine learning, and molecular docking to investigate the potential mechanisms by which ochratoxin A (OTA) contributes to the development of prostate cancer. The findings indicate that OTA might be involved in the onset of prostate cancer through various pathways, such as endocrine disruption, metabolic reprogramming, the regulation of key oncogenic signals, and modulation of the tumor microenvironment.

### 3.1. Key Targets and Molecular Interactions

Machine learning identified five key targets—ESR1, TP53, TNF, INS, and EGFR—indicating that hormonal signaling, tumor suppression, and growth factor pathways might be disrupted.

The occurrence and progression of prostate cancer are closely associated with hormone levels [26]. The molecular docking results indicated a high binding affinity between ESR1 (estrogen receptor α) and OTA, suggesting that OTA might function as an endocrine disruptor by disrupting estrogen signaling. Although no studies have directly confirmed that OTA can activate the estrogen receptor ESR1, OTA can interfere with estrogen signaling by modulating PPARγ-mediated lipid metabolism and disrupting the hypothalamic–pituitary–gonadal axis [27,28]. Gene Ontology (GO) terms related to hormone-stimulated cellular responses further support the role of OTA in disrupting the effects of ESR1. Additionally, the abnormal expression of ESR1 in prostate epithelial cells may promote epithelial–mesenchymal transition (EMT) and extracellular matrix (ECM) remodeling [29,30,31,32], which aligns with our KEGG results concerning ECM–receptor interactions.

TP53 is a crucial tumor suppressor that plays a role in DNA repair and cell cycle regulation [33]. In prostate cancer, mutations in TP53 result in a loss of p53 function, leading to unchecked cell cycle progression and the inhibition of apoptosis [34]. Furthermore, abnormalities in TP53 can modify the tumor microenvironment by impacting inflammatory factors [35,36], aligning with our findings.

TNF is a cytokine involved in inflammation and immune responses. The binding of OTA to TNF may exacerbate chronic inflammation, a known risk factor for cancer [37,38]. KEGG pathways associated with viral infections and natural killer cell cytotoxicity suggest that OTA could modulate immune responses to facilitate tumor progression. GO terms related to cytokine-stimulated responses and collagen activation pathways further corroborate the role of TNF in mediating OTA-induced inflammation.

INS is involved in metabolic regulation and cell growth [39]. The binding of OTA to INS may disrupt insulin signaling, leading to metabolic disorders and increased cell proliferation [40]. Elevated insulin levels can induce chronic low-grade inflammation and oxidative stress, which promote the formation of a tumor microenvironment and enhance the aggressiveness of prostate cancer [41]. The activation of the AGE-RAGE signaling pathway in diabetic complications, as our KEGG results show, further supports the role of INS in mediating OTA-induced metabolic changes.

EGFR is a receptor tyrosine kinase (RTK) that plays a crucial role in cell proliferation, survival, migration, and differentiation [42]. The Isolation Forest algorithm has labeled EGFR as having unique network characteristics, indicating that it may significantly influence the cellular signaling network through multi-target synergistic effects, ultimately contributing to tumorigenesis. The binding of OTA to EGFR can activate downstream signaling pathways, such as the PI3K-Akt signaling pathway, thereby promoting the survival and proliferation of cancer cells [43]. KEGG pathways related to cancer and focal adhesion suggest that the binding of OTA to EGFR may enhance the oncogenic potential of prostate cells [44,45,46,47].

### 3.2. KEGG Pathway Analysis: Significance for Prostate Cancer

The KEGG pathway analysis revealed several significant pathways, including cancer pathways, the PI3K-Akt signaling pathway, focal adhesion, and viral infections. These pathways are interconnected, highlighting the multifaceted nature of OTA-induced carcinogenesis. In particular, the PI3K-Akt signaling pathway, as a central hub integrating signals from various receptors and pathways, leads to increased cell survival, proliferation, and resistance to apoptosis [48,49]. The enrichment of the “focal adhesion” and “ECM–receptor interaction” pathways indicates that OTA may affect the remodeling of the tumor microenvironment, promoting cancer cell invasion and metastasis. The presence of viral-infection-related pathways suggests that OTA may exacerbate inflammation or immune dysregulation, thereby influencing the occurrence and development of prostate cancer. Interestingly, “prostate cancer” itself emerged as a relevant pathway, further supporting the potential mechanistic link between OTA exposure and prostate cancer development.

### 3.3. GO Functional Analysis: Cellular and Molecular Effects

Biological processes related to GO emphasize the potential impact of OTA on cell activation and responses to hormones, cytokines, and protein phosphorylation, all of which are crucial in cancer cell signaling [50]. The strong enrichment of collagen-related pathways, such as “collagen activation signaling”, suggests that OTA may alter ECM dynamics, a key factor in tumor progression and metastasis [51,52], highlighting the importance of the cellular environment in mediating OTA-induced effects. At the molecular function level, OTA appears to affect protein kinase activity, particularly tyrosine kinases (such as EGFR) and tissue-modifying enzymes, which may lead to abnormal gene expression and epigenetic changes in cancer cells [53], further emphasizing the role of signaling pathways in OTA-induced carcinogenesis.

### 3.4. Implications for Prostate Cancer Subtypes

Although this study did not explicitly establish models for androgen-dependent and -independent prostate cancer, multiple observations suggest that the mechanisms of action of OTA may span different disease stages. TP53 dysfunction is a typical feature of metastatic castration-resistant prostate cancer (mCRPC) [34]. EGFR- and INS-mediated activation of the PI3K-Akt signaling pathway can partially compensate for the loss of androgen receptor (AR) signaling, particularly in the PTEN-deficient subtype of castration-resistant prostate cancer (CRPC) [54]. The upregulation of ESR1 is associated with AR-negative neuroendocrine differentiation [55], which is consistent with the predicted estrogenic disruption effects of OTA. However, the relative contributions of OTA in the early (hormone-sensitive) and late (castration-resistant) stages remain speculative. Future studies should compare the effects of OTA in AR-positive and -negative cell lines and assess OTA exposure in the tissue models of patients with castration-resistant prostate cancer.

These findings not only elucidate the mechanisms through which OTA promotes cancer development but also underscore its considerable impact on public health. We must concentrate on the levels of dietary OTA exposure within the population, particularly in foods that are susceptible to OTA contamination, such as coffee and cereals. Furthermore, considering that the OTA content in foods more commonly consumed by men, such as beer and coffee, might be underestimated, a reevaluation of their potential risks to prostate health is advised. The outcomes of this study advocate for the inclusion of prostate cancer risk in the food safety risk assessment system for OTA and offer a scientific foundation for the creation of targeted dietary intervention strategies and regulatory measures.

However, this study has several limitations. First, as a computational study, all predictions may be subject to algorithmic biases, incomplete database coverage, and other uncertainties. Second, neither species-specific toxicokinetic variations nor individual genetic polymorphisms that may influence susceptibility were assessed. Additionally, the potential toxicity of OTA metabolites has not yet been considered. Future studies should further validate these findings through animal experiments and single-cell sequencing technologies.

## 4. Conclusions

In summary, through the integration of various technical approaches, this study elucidated that OTA technology may promote the development of prostate cancer via the following pathways: endocrine disruption (involving ESR1 and hormone response pathways); the activation of oncogenic signaling (specifically, the PI3K-Akt signaling pathway); metabolic reprogramming (through the AGE-RAGE signaling pathway); and modulation of the tumor microenvironment (including ECM remodeling and collagen signaling). These mechanisms may interact with each other, collectively promoting the progression of prostate cancer.

## 5. Methods

### 5.1. Prediction of Ochratoxin A Toxicity

The SMILES information for OTA was retrieved from the PubChem database (https://pubchem.ncbi.nlm.nih.gov). ProTox-3.0 (https://tox.charite.de/protox3/) was utilized to predict OTA’s toxicological properties based on its chemical structure. The model ensemble was executed under default settings, with predictions with a probability threshold of ≥0.7 considered reliable and included for further analysis.

### 5.2. Target Screening of Ochratoxin A

We first obtained OTA’s molecular structure from PubChem and then searched it against the ChEMBL (https://www.ebi.ac.uk/chembl) and SwissTargetPrediction (http://www.swisstargetprediction.ch) databases, limiting the species to Homo sapiens. After systematically compiling and deduplicating the potential targets, we used UniProt (https://www.uniprot.org) to standardize the target nomenclature, ultimately building an OTA-specific target library.

### 5.3. Target Screening of Prostate Cancer

Prostate-cancer-related candidate targets were retrieved from the GeneCards database (http://www.genecards.org) using the keywords “prostate cancer” and “prostate tumor”. The entries were ranked based on their relevance score, and the top 5000 hits for each keyword were selected. This threshold captured the majority of high-confidence, disease-associated genes while minimizing low-relevance noise, thus balancing computational feasibility with biological significance. Including additional genes could inflate false-positive associations, whereas a stricter cutoff might exclude potentially important targets [56]. After merging the two result sets, duplicate entries were removed to establish a comprehensive target gene set.

### 5.4. Common Targets

Using a Venn diagram [57], we identified the intersection between the targets of OTA and those related to prostate cancer. These common targets were determined as the potential targets through which OTA may induce prostate cancer.

### 5.5. Protein–Protein Interaction Analysis

The common targets were imported into the STRING database (https://string-db.org) with the following filters: (1) species restricted to Homo sapiens; (2) medium confidence (≥0.4); (3) all evidence channels retained to maximize sensitivity; and (4) hidden disconnected nodes in the network removed. These parameters were selected to balance network completeness with analytical specificity.

The resulting protein–protein interaction (PPI) network was constructed and visualized using Cytoscape (version 3.9.1). A network topology analysis was performed with the following parameters: (1) nodes with a degree centrality of at least 5 were considered hub targets; (2) betweenness and closeness centrality values were calculated using the NetworkAnalyzer tool with default parameters; and (3) the MCODE plugin was used for a cluster analysis with default parameters (degree cutoff = 2, node score cutoff = 0.2, k-core = 2, and max depth = 100).

### 5.6. Gene Ontology (GO) Functional and Kyoto Encyclopedia of Genes and Genomes (KEGG) Pathway Enrichment Analyses

For the common targets of OTA and prostate cancer, we performed GO functional annotation and KEGG pathway enrichment analyses using the Metascape database (http://metascape.org). Based on functional importance, pathway centrality, and relevance to prostate cancer, we selected the top 20 most significant KEGG pathways and generated a histogram for the enrichment analysis. Building on the enrichment results, we generated a Sankey diagram to map the functional associations between genes and their related pathways.

### 5.7. Machine Learning Workflow and Identification of Potential Core Targets

To systematically identify the core targets, we implemented a machine learning pipeline. All computations were performed in Python (Version: 3.9) using the open-source libraries scikit-Learn (Version: 2.2.3), pandas (Version: 2.2.3), matplotlib (Version: 3.10.1), and numPy (Version: 2.2.5); the random seed was fixed at 42 to ensure full reproducibility.

#### 5.7.1. Feature Engineering

Fourteen topological indices exported from the CytoNCA plug-in (MNC, EPC, Bottleneck, BetweennessCentrality, ClosenessCentrality, Degree, Radiality, Stress, AverageShortestPathLength, ClusteringCoefficient, NeighborhoodConnectivity, TopologicalCoefficient, NumberOfUndirectedEdges, and Betweenness) served as the raw feature set. Features with <0.5% missing values were imputed with the mean; samples with ≥0.5% missing values were removed. All features were then Z-score-standardized using StandardScaler (mean = 0, SD = 1) to remove any scale differences.

#### 5.7.2. Model Selection and Hyper-Parameters

(1) K-means clustering: Euclidean distance, scikit-learn KMeans, max_iter = 300, and tol = 1 × 10^−4^. The optimal cluster number was determined using the elbow method: WCSS was computed for k = 1–10; the second-difference maximum indicated an elbow at k = 3 (Figure 7).
(2) Isolation Forest anomaly detection [19]: scikit-learn IsolationForest, n_estimators = 100, subsample = 0.8, and contamination = 0.10 (an empirically chosen value commonly used in [19]).
(3) PCA visualization: the first two principal components (n_components = 2) were retained.

#### 5.7.3. Composite Score Construction

For each gene g and topological index i, Z-scores z_g,i were calculated. The indices were then ranked according to their biological interpretation, as follows:

(1) Centrality indices in which higher values indicate importance (11 indices, e.g., MNC, EPC, and Betweenness) were ranked in descending order;
(2) Path-based indices in which lower values indicate importance (3 indices, e.g., AverageShortestPathLength and TopologicalCoefficient) were ranked in ascending order.

The composite score for gene g is the arithmetic mean of its ranks across all valid indices, as follows:

Composite_score_g = (1/|I_valid|) Σ_i∈I_valid R_g,i

where I_valid is the set of successfully calculated indices (|I_valid| = 14). Genes were finally re-ranked based on their Composite_score_g in ascending order; lower scores indicate a higher overall importance.

#### 5.7.4. Integration and Core Gene Definition

Integrating cluster labels, anomaly flags, and composite ranks, a gene was designated as a core gene if it simultaneously satisfied the following conditions:

(1) IsolationForest anomaly flag = −1 (Is_outlier = 1);
(2) Composite_rank ≤ 20.

## Figures and Tables

**Table 1:** OTA toxicity prediction using ProTox-3.0.

Target	Prediction	Probability
Cytotoxicity	Active	0.99
Clinical toxicity	Active	0.78
Respiratory toxicity	Active	0.73
Nephrotoxicity	Active	0.72
Carcinogenicity	Active	0.71
Immunotoxicity	Inactive	0.97
Mutagenicity	Inactive	0.92
BBB-barrier	Inactive	0.68
Hepatotoxicity	Inactive	0.65
Cardiotoxicity	Inactive	0.65
Nutritional toxicity	Inactive	0.64
Ecotoxicity	Inactive	0.60
Neurotoxicity	Inactive	0.54

**Table 2:** Top 10 potential key genes based on topological metrics.

Gene	Composite Score
TP53	4.14
TNF	4.71
INS	6.14
EGFR	6.18
ESR1	6.86
MMP9	7.53
ITGB1	8.46
RHOA	9.25
ATM	9.89
UBA52	10.60

## Data Availability

The original contributions presented in this study are included in this article/its Supplementary Materials. Further inquiries can be directed to the corresponding author.

## Supplementary Materials

The following supporting information can be downloaded at: https://www.mdpi.com/article/10.3390/toxins17080388/s1.
