# Supplementary material for: The Potential Mechanisms of Ochratoxin A in Prostate Cancer Development: An Integrated Study Combining Network Toxicology, Machine Learning, and Molecular Docking
Source: Toxins (Basel). 2025 Aug 4;17(8):388. doi: 10.3390/toxins17080388 (PMC12389974; doi:10.3390/toxins17080388)
Supplement: Supplementary file 1 [file toxins-17-00388-s001.zip › hub gene/1.docx]

Top 5 Prostate Cancer-Related Genes (Based on Random Forest Probability):

Gene Probability

0 TP53 0.881810

1 TNF 0.881810

3 INS 0.832310

4 ESR1 0.685643

2 EGFR 0.487643

Top 5 Prostate Cancer-Related Genes (Based on Gradient Boosting Probability):

Gene Probability

0 TP53 0.701049

1 TNF 0.701049

3 INS 0.701049

4 ESR1 0.701049

2 EGFR 0.066843
