# Supplementary figures and images for: The Potential Mechanisms of Ochratoxin A in Prostate Cancer Development: An Integrated Study Combining Network Toxicology, Machine Learning, and Molecular Docking
Source: Toxins (Basel). 2025 Aug 4;17(8):388. doi: 10.3390/toxins17080388 (PMC12389974; doi:10.3390/toxins17080388)

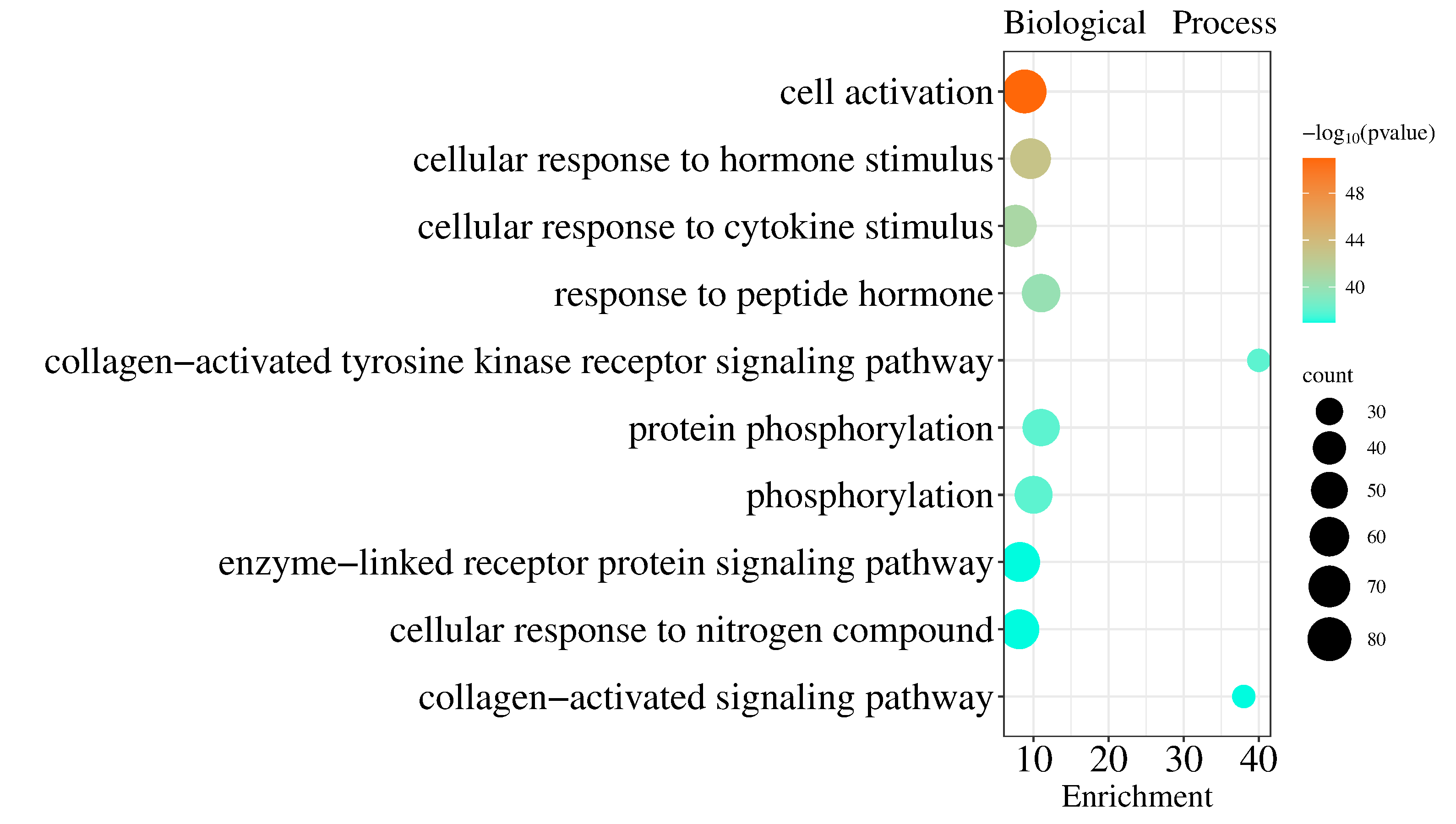

Supplement: Supplementary file 1 [file toxins-17-00388-s001.zip › GO and KEGG/BP.png]

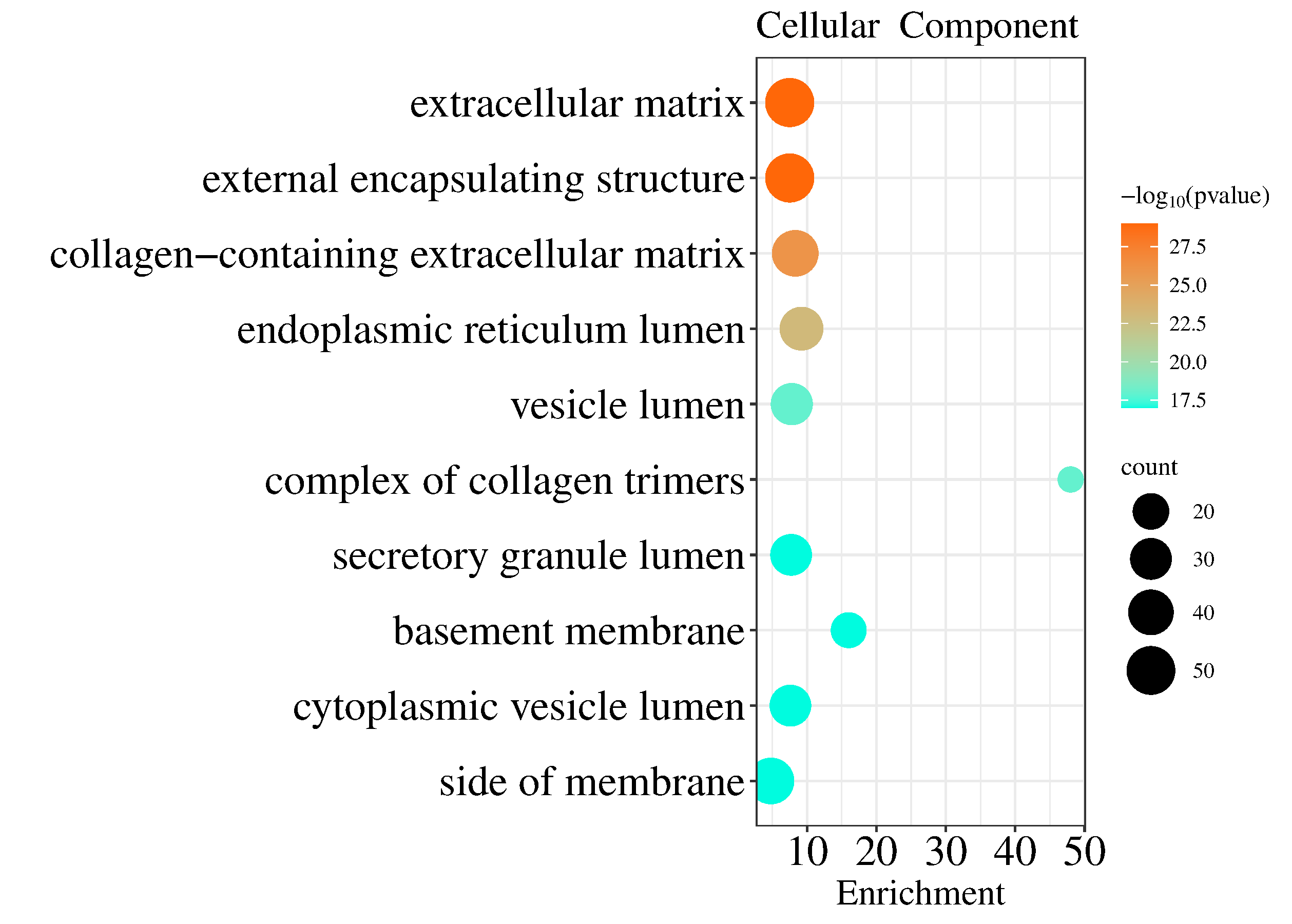

Supplement: Supplementary file 1 [file toxins-17-00388-s001.zip › GO and KEGG/CC.png]

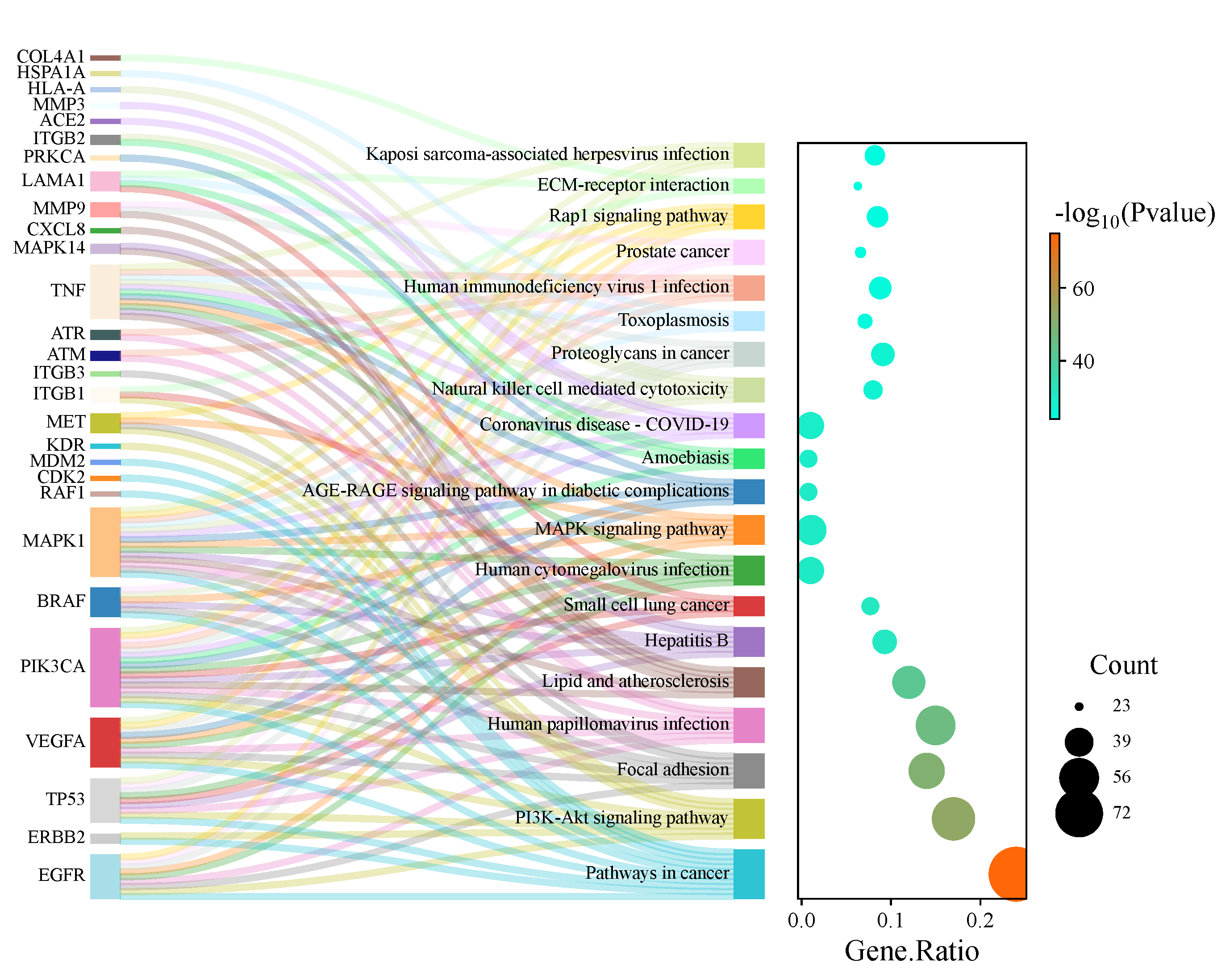

Supplement: Supplementary file 1 [file toxins-17-00388-s001.zip › GO and KEGG/KEGG 桑吉.png]

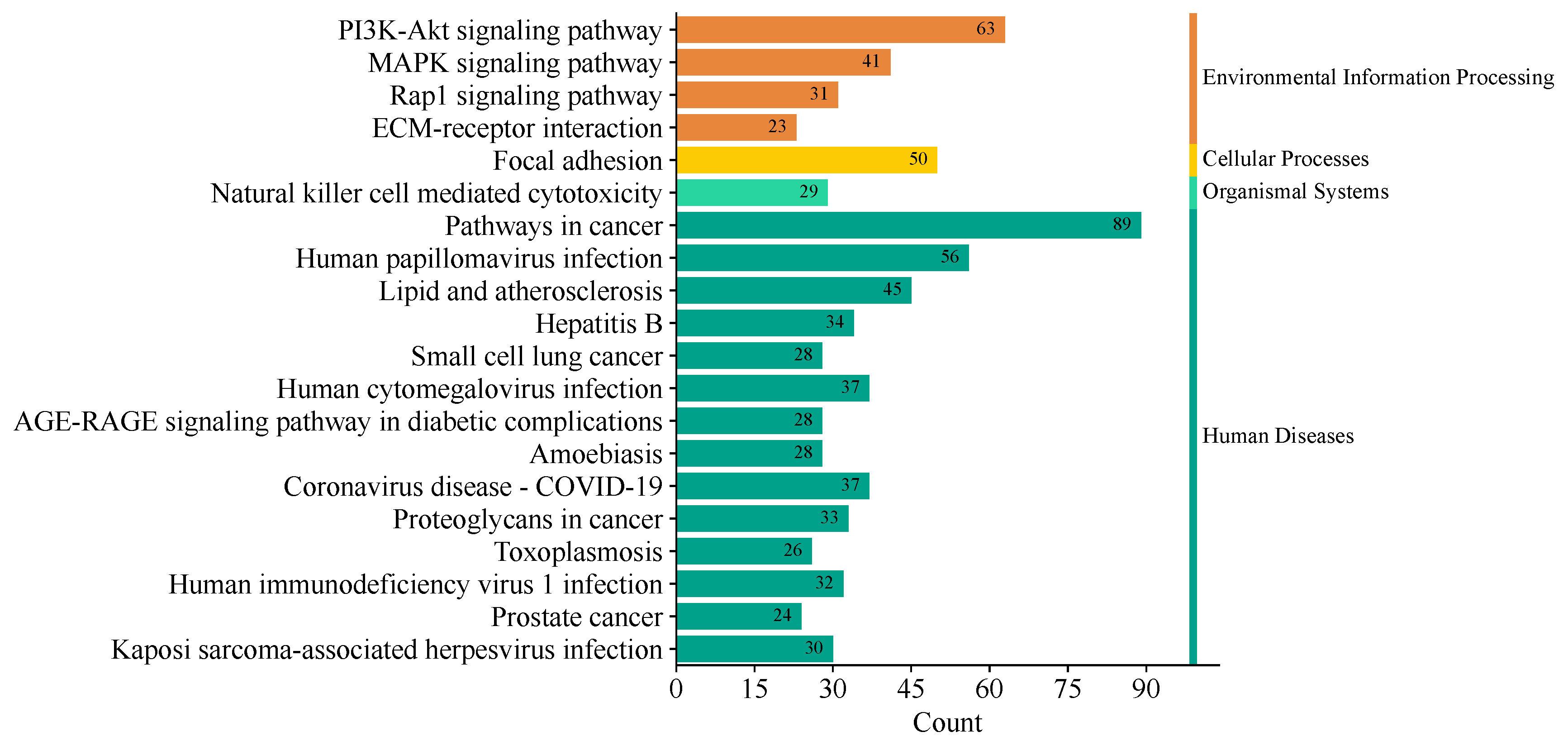

Supplement: Supplementary file 1 [file toxins-17-00388-s001.zip › GO and KEGG/KEGG.png]

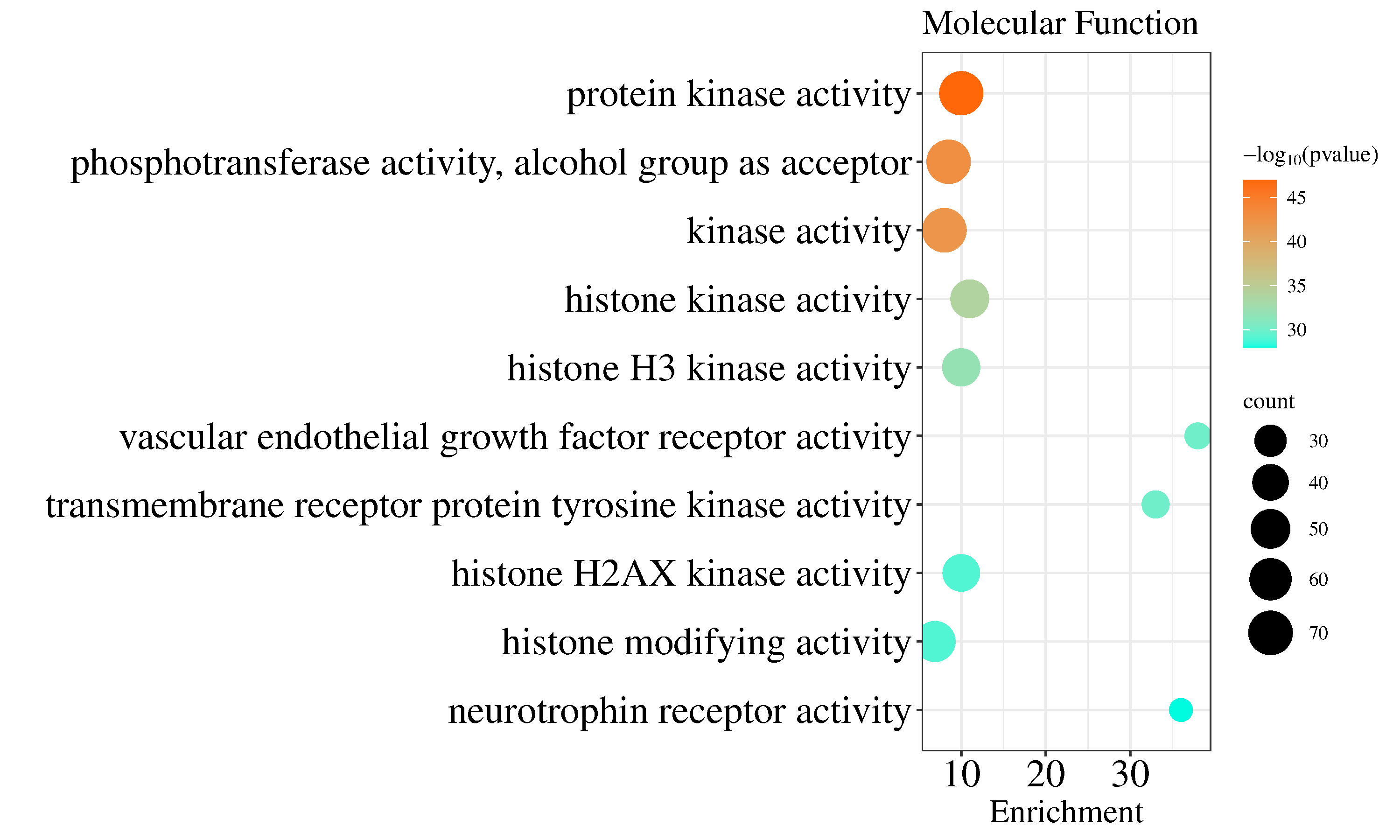

Supplement: Supplementary file 1 [file toxins-17-00388-s001.zip › GO and KEGG/MF.png]

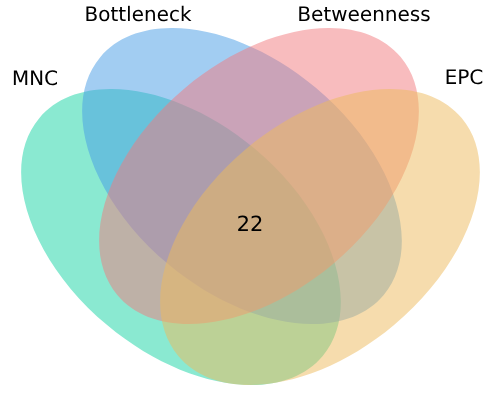

Supplement: Supplementary file 1 [file toxins-17-00388-s001.zip › hub gene/jVenn_chart.png]

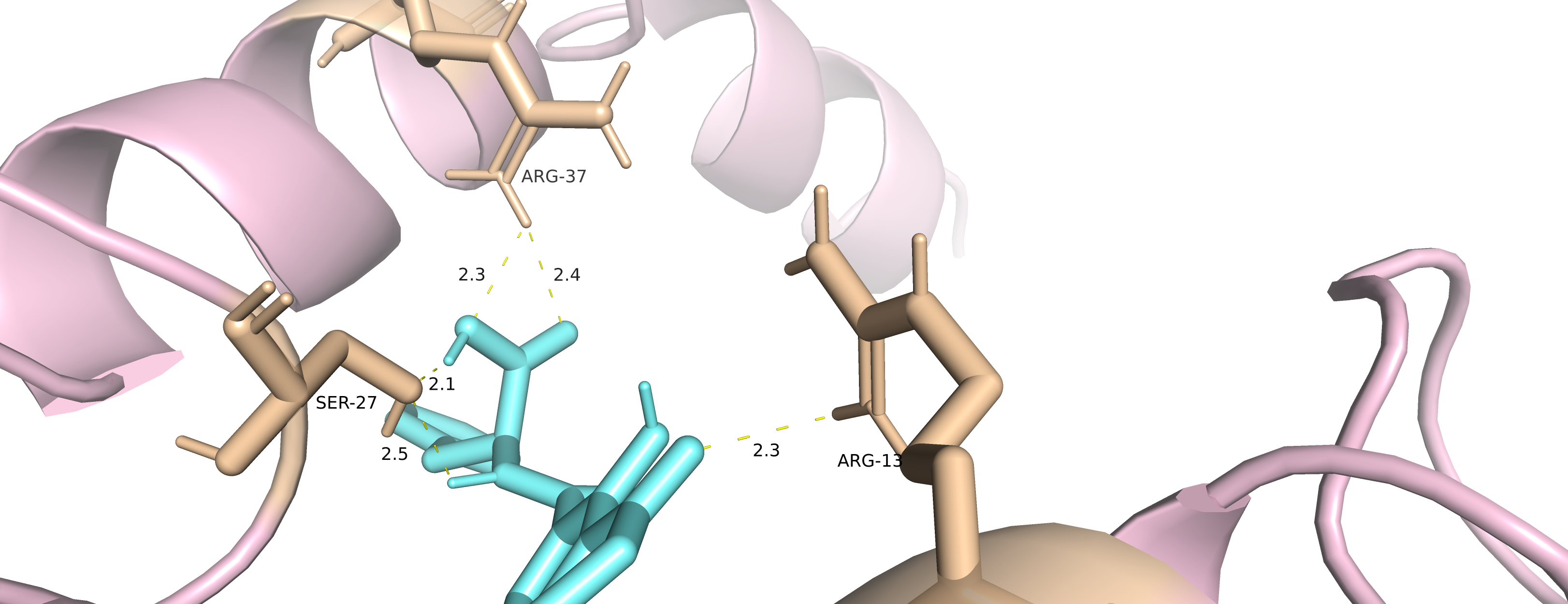

Supplement: Supplementary file 1 [file toxins-17-00388-s001.zip › molecular docking/EGFR/EGFR.png]

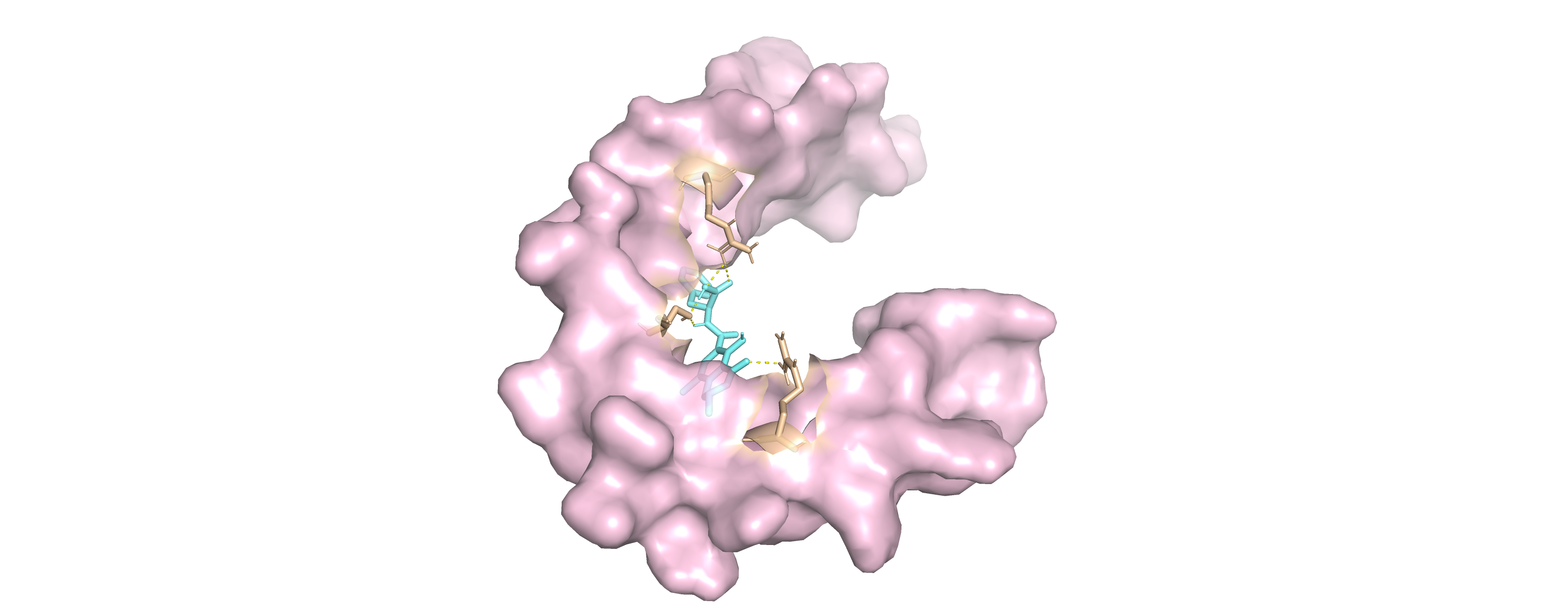

Supplement: Supplementary file 1 [file toxins-17-00388-s001.zip › molecular docking/EGFR/EGFRa.png]

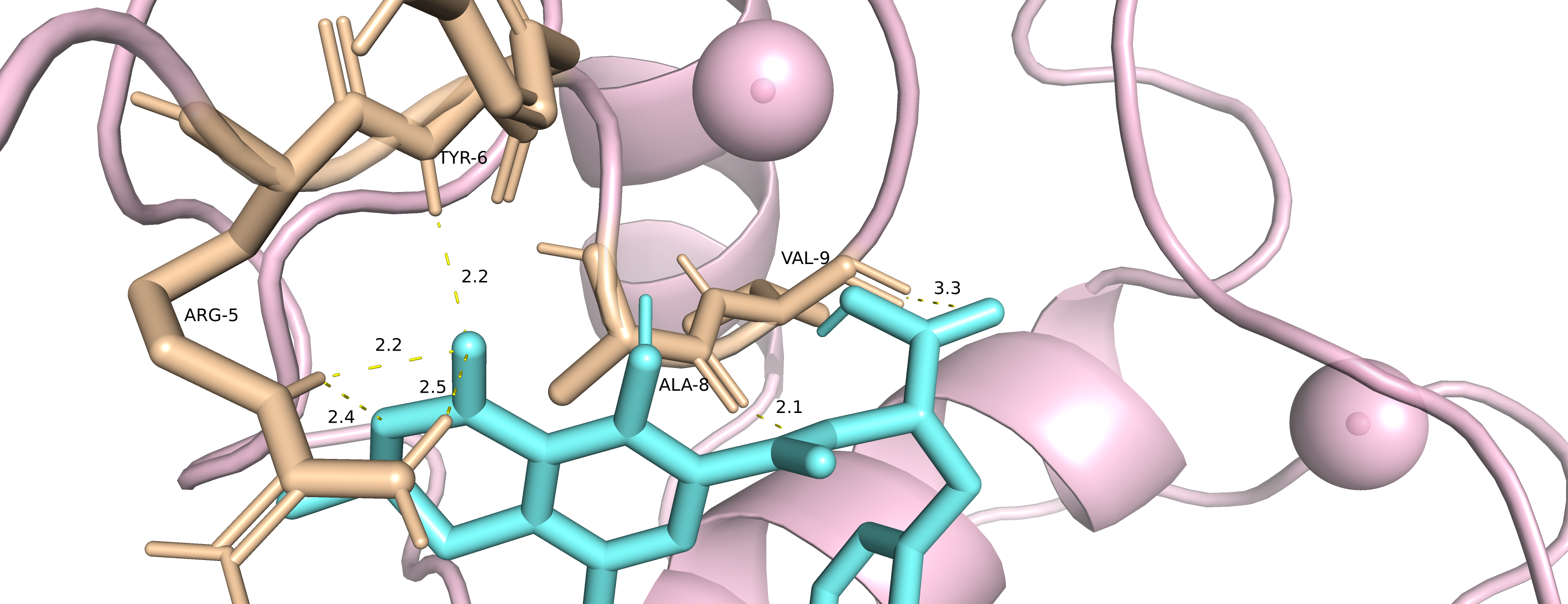

Supplement: Supplementary file 1 [file toxins-17-00388-s001.zip › molecular docking/ESR1/ESR1.png]

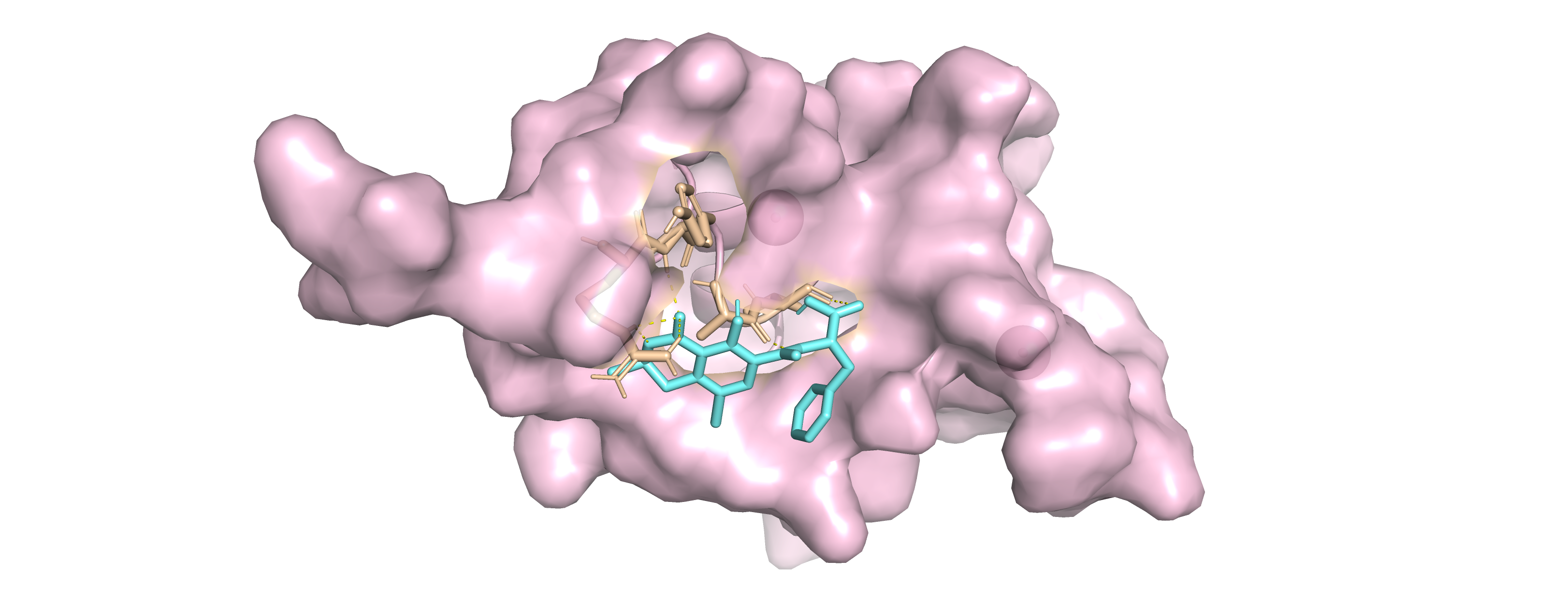

Supplement: Supplementary file 1 [file toxins-17-00388-s001.zip › molecular docking/ESR1/ESR11.png]

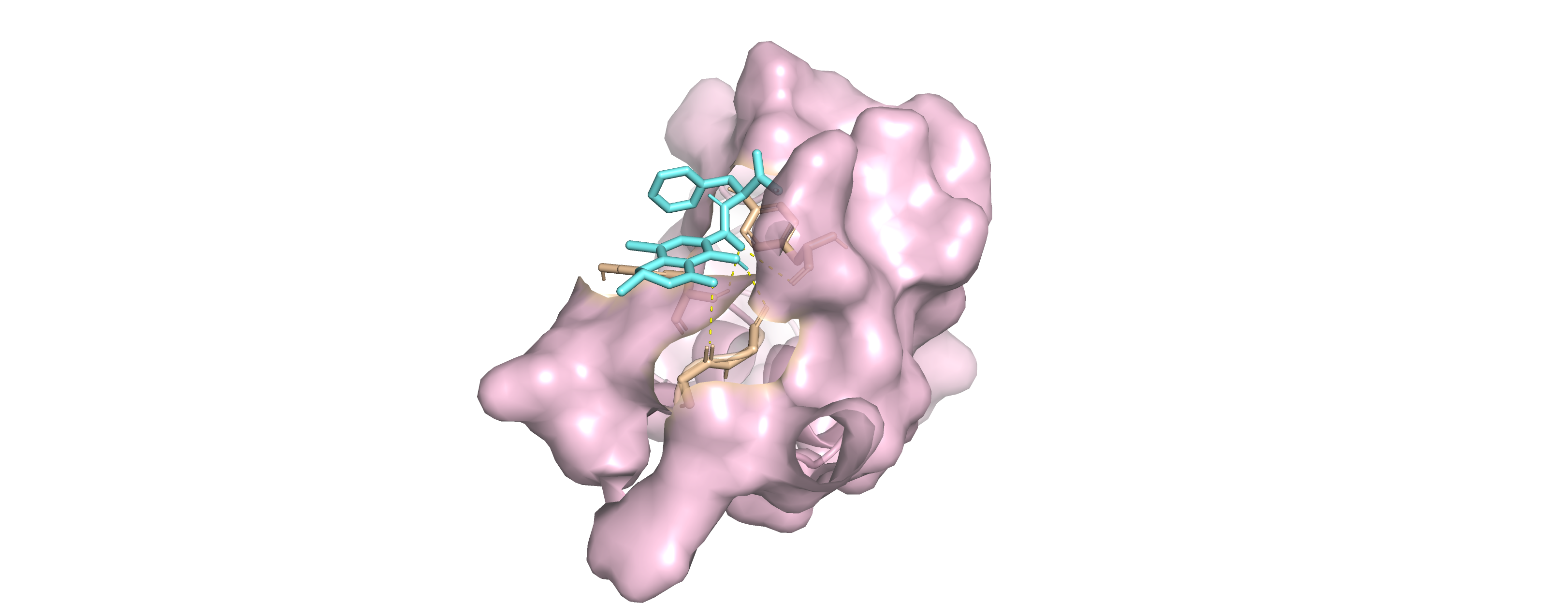

Supplement: Supplementary file 1 [file toxins-17-00388-s001.zip › molecular docking/INS/ins.png]

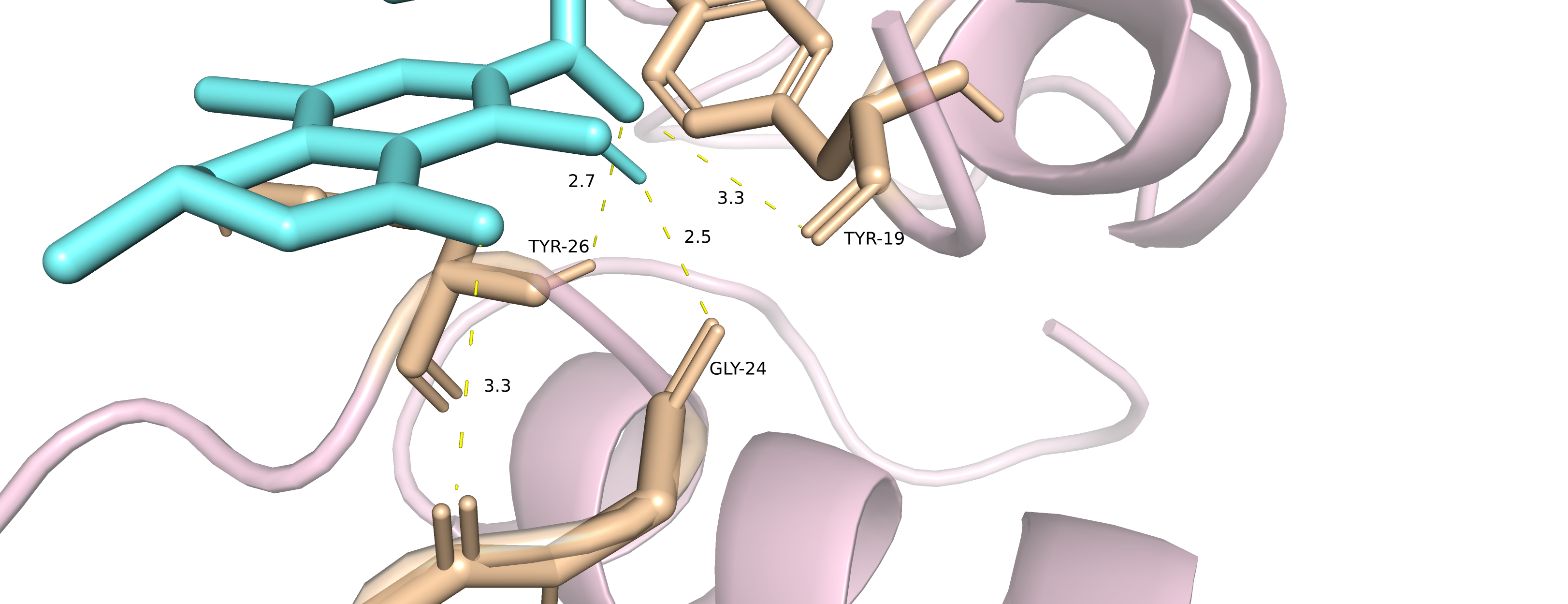

Supplement: Supplementary file 1 [file toxins-17-00388-s001.zip › molecular docking/INS/ins1.png]

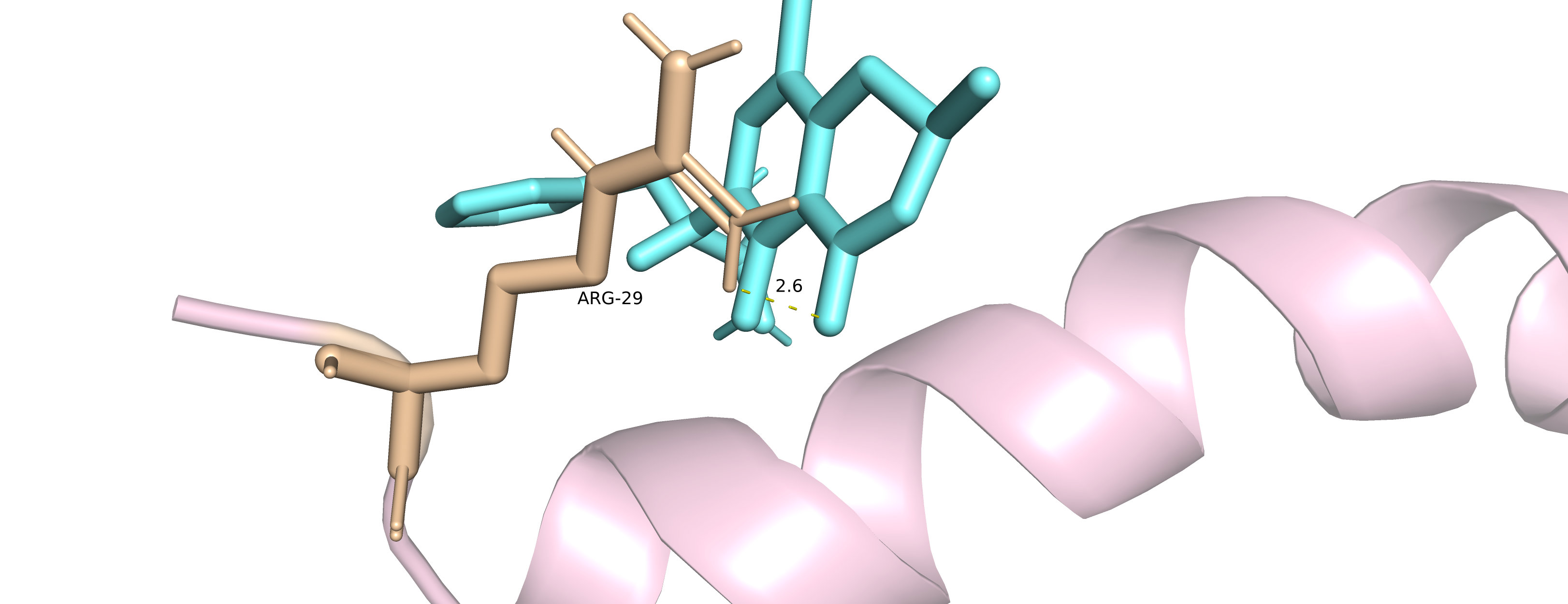

Supplement: Supplementary file 1 [file toxins-17-00388-s001.zip › molecular docking/TNF/TNF.png]

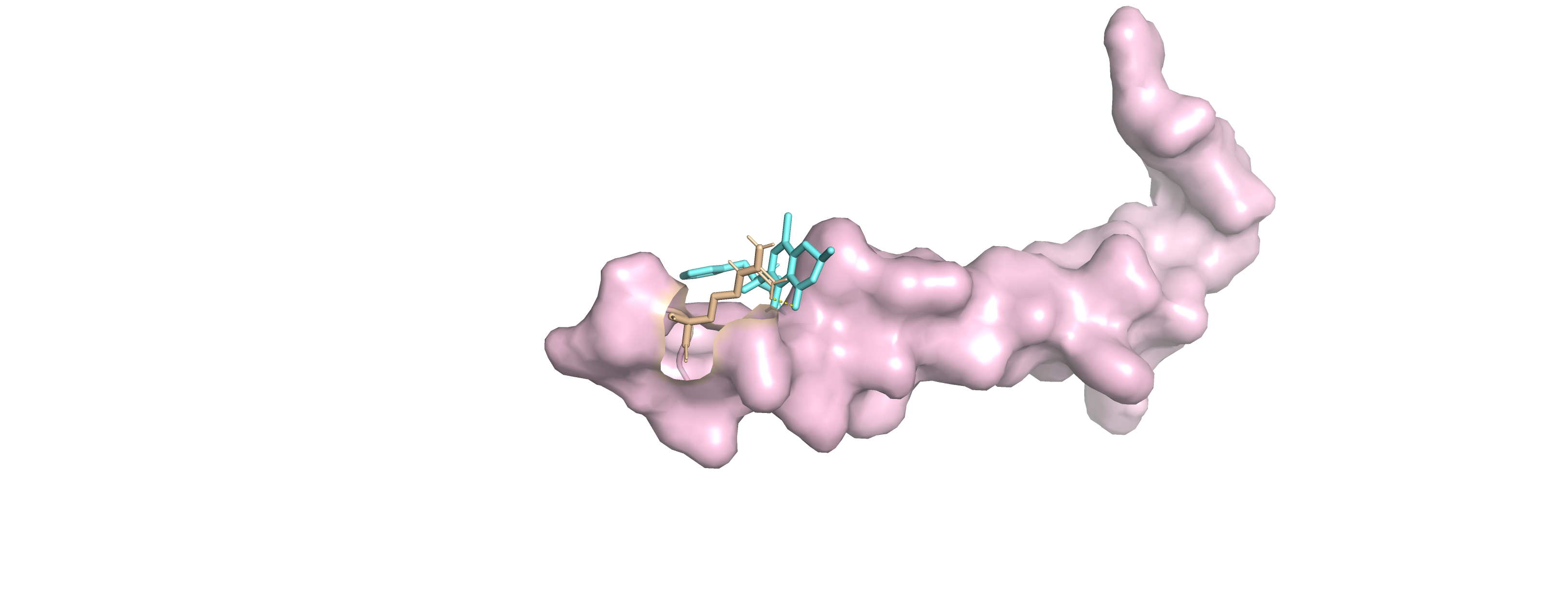

Supplement: Supplementary file 1 [file toxins-17-00388-s001.zip › molecular docking/TNF/TNFa.png]

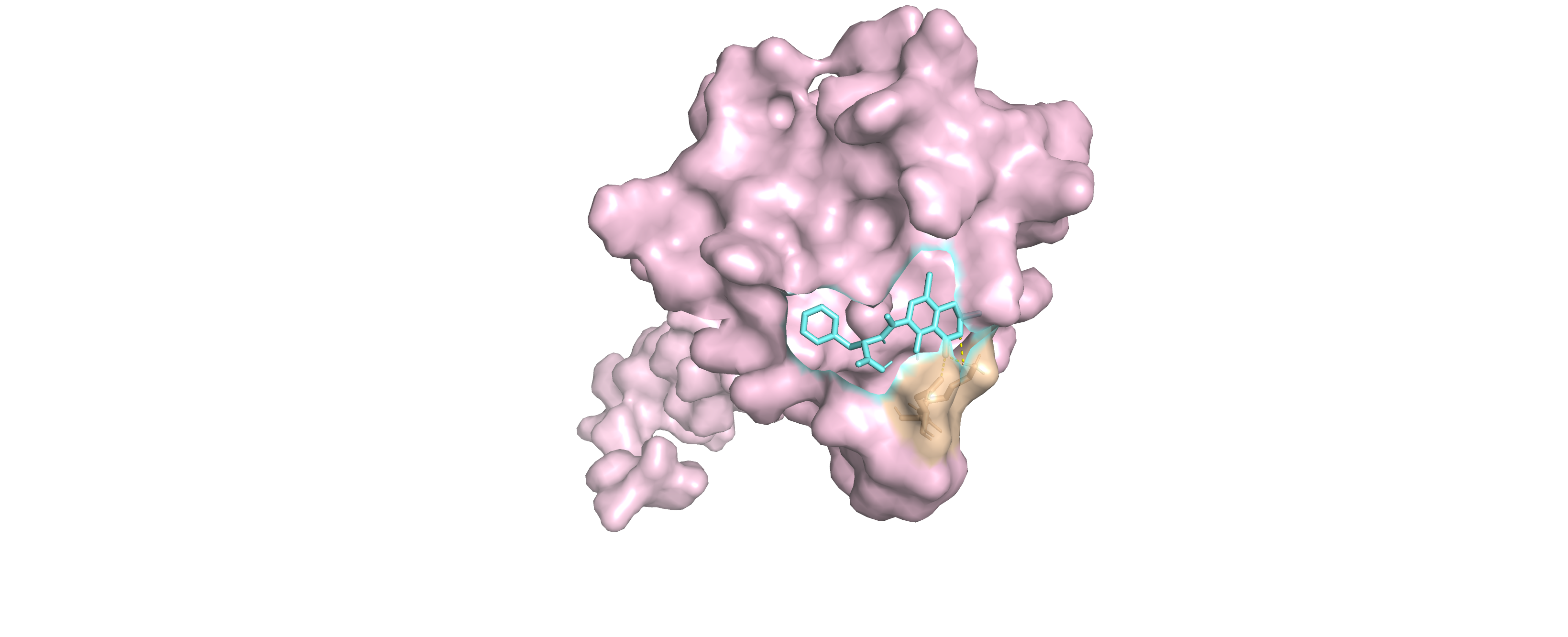

Supplement: Supplementary file 1 [file toxins-17-00388-s001.zip › molecular docking/TP53/TP53.png]

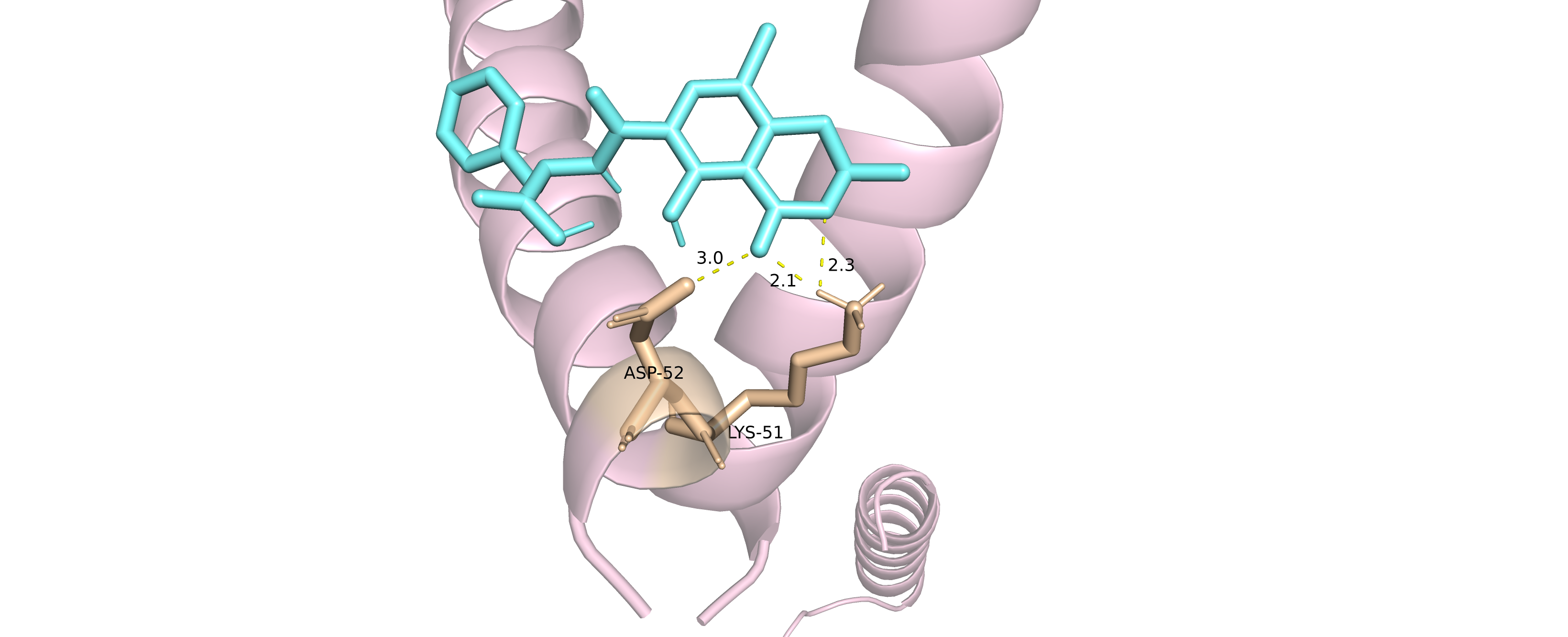

Supplement: Supplementary file 1 [file toxins-17-00388-s001.zip › molecular docking/TP53/TP53A.png]

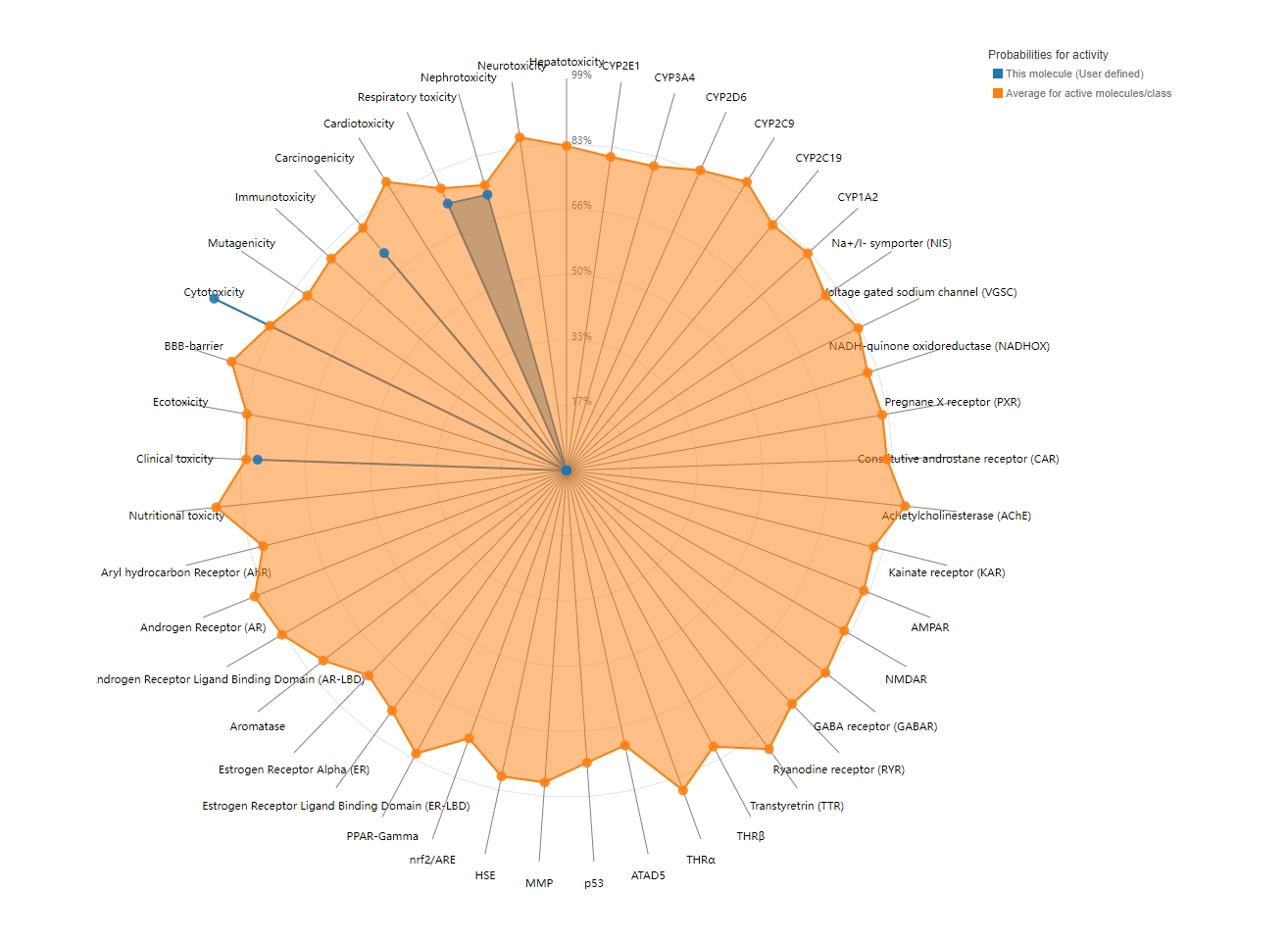

Supplement: Supplementary file 1 [file toxins-17-00388-s001.zip › OTA chemical structure/OTA tox.png]

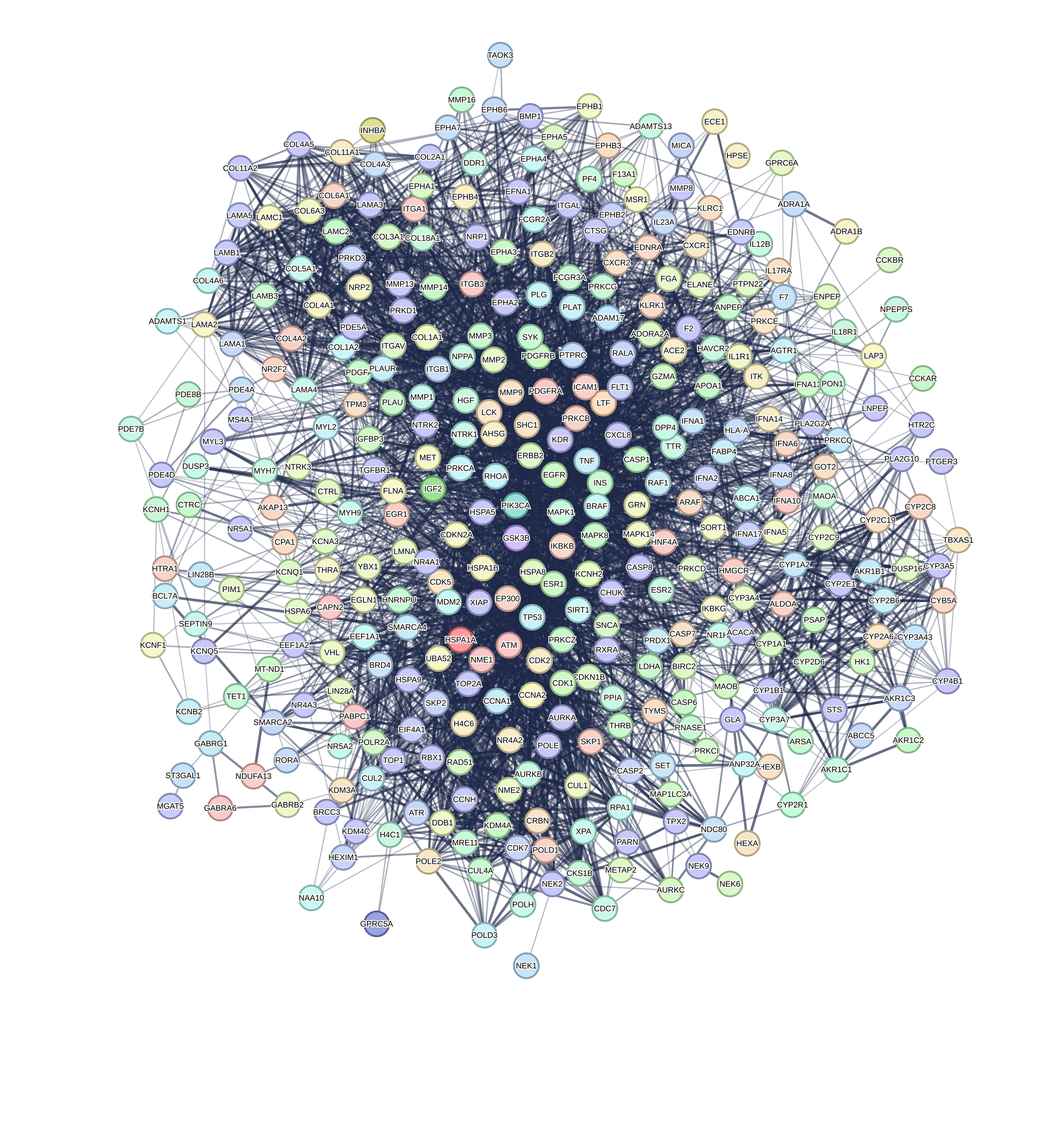

Supplement: Supplementary file 1 [file toxins-17-00388-s001.zip › PPI/string_hires_image (1).png]

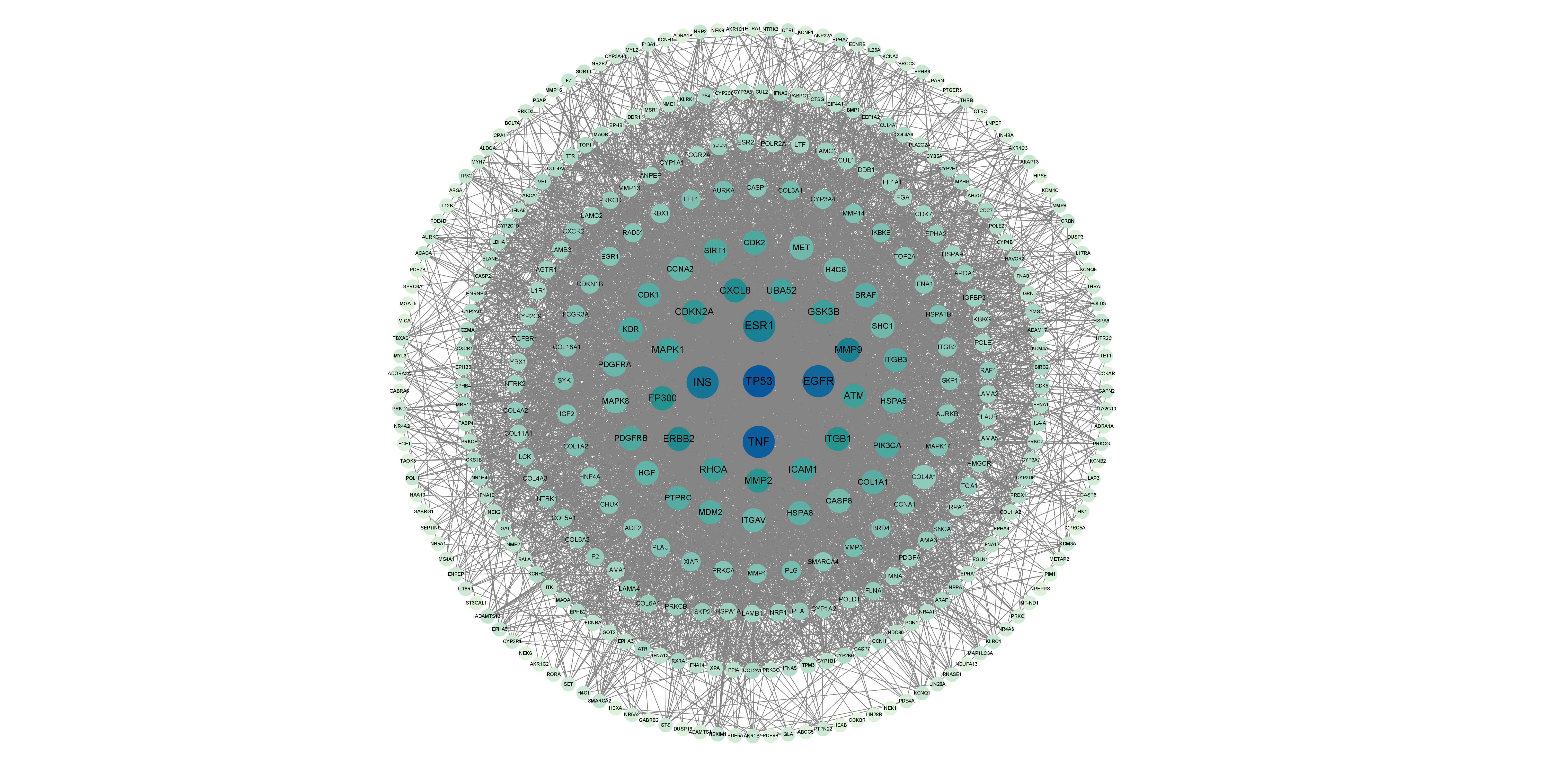

Supplement: Supplementary file 1 [file toxins-17-00388-s001.zip › PPI/string_interactions_short (1).tsv.png]

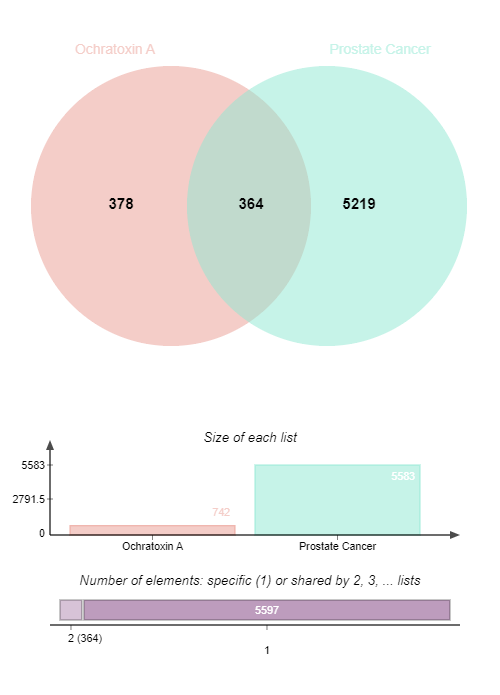

Supplement: Supplementary file 1 [file toxins-17-00388-s001.zip › Target intersection/jVenn_chart.png]
